# Disease severity and status in Stevens–Johnson syndrome and toxic epidermal necrolysis: Key knowledge gaps and research needs

**DOI:** 10.3389/fmed.2022.901401

**Published:** 2022-09-12

**Authors:** Rannakoe J. Lehloenya

**Affiliations:** ^1^Division of Dermatology, Department of Medicine, University of Cape Town, Cape Town, South Africa; ^2^Combined Drug Allergy Clinic, Groote Schuur Hospital, Cape Town, South Africa

**Keywords:** Stevens–Johnson syndrome (SJS) and toxic epidermal necrolysis (TEN) (SJS/TEN), severity, internal organ involvement, depth, knowledge gaps

## Abstract

Stevens–Johnson syndrome and toxic epidermal necrolysis (SJS/TEN) are on a spectrum of cutaneous drug reactions characterized by pan-epidermal necrosis with SJS affecting < 10% of body surface area (BSA), TEN > 30%, and SJS/TEN overlap between 10 and 30%. Severity-of-illness score for toxic epidermal necrolysis (SCORTEN) is a validated tool to predict mortality rates based on age, heart rate, BSA, malignancy and serum urea, bicarbonate, and glucose. Despite improved understanding, SJS/TEN mortality remains constant and therapeutic interventions are not universally accepted for a number of reasons, including rarity of SJS/TEN; inconsistent definition of cases, disease severity, and endpoints in studies; low efficacy of interventions; and variations in treatment protocols. Apart from mortality, none of the other endpoints used to evaluate interventions, including duration of hospitalization, is sufficiently standardized to be reproducible across cases and treatment centers. Some of the gaps in SJS/TEN research can be narrowed through international collaboration to harmonize research endpoints. A case is made for an urgent international collaborative effort to develop consensus on definitions of endpoints such as disease status, progression, cessation, and complete re-epithelialization in interventional studies. The deficiencies of using BSA as the sole determinant of SJS/TEN severity, excluding internal organ involvement and extension of skin necrosis beyond the epidermis, are discussed and the role these factors play on time to healing and mortality beyond the acute stage is highlighted. The potential role of artificial intelligence, biomarkers, and PET/CT scan with radiolabeled glucose as markers of disease status, activity, and therapeutic response is also discussed.

## Background

Stevens–Johnson syndrome (SJS) and toxic epidermal necrolysis (TEN), collectively referred to as epidermal necrolysis (SJS/TEN), are on a spectrum of the same life-threatening drug reaction. The primary feature of SJS/TEN is pan-epidermal necrosis of the skin and mucous membranes. In SJS, there is < 10% of body surface area (BSA) with epidermal detachment while in TEN there is > 30%. SJS/TEN overlap lies between these two extremes (1). TEN is considered the more severe phenotype and is associated with significantly higher mortality of up to 40% (2). The severity-of-illness score for toxic epidermal necrolysis (SCORTEN) is currently the most widely used validated tool to predict mortality rates, although its accuracy has been questioned in certain settings and alternative scores developed (3, 4). SCORTEN predictors of higher mortality in acute settings are age > 40 years, heart rate > 120 bpm, BSA > 10%, serum urea > 10 mmol/L, serum bicarbonate < 20 mmol/L, serum glucose > 14 mmol/L, and cancer or hematological malignancies (3).

Despite improved understanding of SJS/TEN in the last 30 years, mortality has remained constant despite global efforts to find effective pharmacotherapeutic interventions (5, 6). These efforts have been hampered, among others, by rarity of SJS/TEN; inconsistent gold-standard definition of cases; inconsistent and inadequate definition of disease severity; inconsistent and inadequate definition of endpoints and clinical outcomes in studies; low clinical effectiveness of current interventions, making it difficult to conduct sufficiently powered studies; and variations in treatment protocols (5–7). A survey of North American clinicians managing SJS/TEN concluded that the length of time before cessation of disease progression and the length of time to complete re-epithelialization are some of the minimum required variables for researchers and clinicians to effectively evaluate SJS/TEN treatment efficacy in a clinically meaningful way. These, as well as mortality and duration of hospitalization, are the endpoints currently used to evaluate pharmacotherapeutic efficacy and other interventions (8). Apart from mortality, none of the others has been standardized sufficiently to be used with reproducible accuracy across individual cases and treatment centers (7).

A systematic review published in March 2022 that included only highest quality studies, namely, randomized-controlled trials and prospective observational comparative studies, found no evidence to support superiority of the following interventions when compared head to head: corticosteroids vs. no corticosteroids; intravenous immunoglobulins (IVIGs) vs. no IVIGs; and cyclosporine vs. IVIGs. However, the study reported a possible reduction in mortality with the use of the TNF-alpha inhibitor etanercept compared to corticosteroids.

The authors assessed three of the four studies included in the comparisons to have very low-certainty evidence and one to have low-certainty evidence. Time to complete re-epithelialization, length of hospital stay, and adverse effects leading to discontinuation of therapy were not reported in the majority of studies. There were no studies that compared etanercept vs. cyclosporine, etanercept vs. IVIG, IVIG vs. supportive care, IVIG vs. cyclosporine, and cyclosporine vs. corticosteroids (7). Another systematic review with a meta-analysis and meta-regression of observational studies also published in March 2022 concluded that the use of etanercept resulted in the lowest mortality rate and the highest IVIG compared to supportive care and other systemic therapies used in SJS/TEN. Corticosteroids were associated the shortest time for re-epithelialization and the shortest length of hospital stay. The authors highlight that the severity of disease seems to influence the choice of therapy by the treating physicians (9). A systematic review and meta-analysis published a few months before these two concluded that systemic glucocorticoids showed a survival benefit for patients with SJS/TEN in all analyses compared with other forms of treatment (10). A common problem highlighted in all these reviews is the heterogeneity of the studies and low confidence in their reproducibility. All conclude that better-designed prospective studies are needed. Despite these challenges, there is more emerging evidence to suggest that combination therapy of etanercept and corticosteroids or etanercept as monotherapy reduces mortality, skin healing time, and hospital stay compared to IVIG combined with corticosteroids or corticosteroid monotherapy (11–13).

The relative rarity of SJS/TEN, and to a lesser extent, the efficacy of current interventions are the two factors that are beyond the immediate control of researchers in the field. Case definition has improved over the years, allowing differentiation from other blistering disorders like erythema multiforme and bullous-fixed drug eruptions (1, 5, 14). The other variables that are inconsistently evaluated and reported in interventional studies for SJS/TEN are amenable to harmonization by a well-directed, focused, and collaborative global effort. Global collaboration, sharing of ideas, and directing research efforts on SJS/TEN are already underway. These international collaborations are an ideal platform to address these issues (15, 16). In this article, research gaps and unmet needs in SJS/TEN research that impact uniformity and consistency in studies that assess therapeutic interventions are highlighted. Also in focus are gaps relating to disease severity, disease status, disease progression or cessation of progression during the acute stage, and definition of disease resolution. Potential future research directions are suggested to address some of these gaps.

## Research gaps

### Body surface area as the sole determinant of severity

Body surface area has an important, validated, and clinically obvious association with in-hospital and early mortality (17, 18).

However, there is considerable evidence showing that BSA impacts mortality only in the first 90 days of SJS/TEN, and that increased mortality is recorded among survivors for up to a year after the acute episode (19). This suggests that factors other than the BSA influence the severity and natural history of SJS/TEN.

### Extension of skin necrosis beyond the epidermis as an additional marker of severity

**FIGURE 1:**
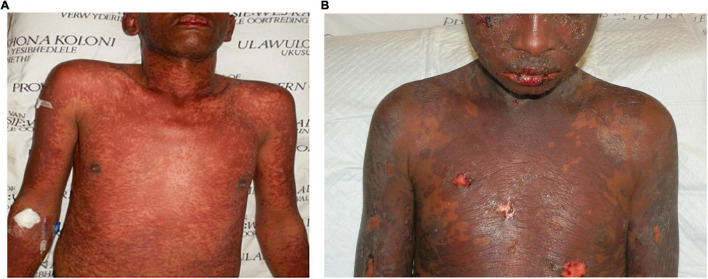
Toxic epidermal necrolysis affecting 40% body surface area in two patients: **(A)** a more superficial variant without denudation of the skin and **(B)** a variant with positive Nikolsky sign and denudation of the skin as well as frank bleeding.

The extension of tissue damage beyond the epidermis by the pathogenic factors involved in SJS/TEN, even in the absence of complications like skin infection, although largely unappreciated currently, seems to impact the time to complete re-epithelialization regardless of treatment approaches taken. Over the years, in our unit we have encountered “definite” cases of TEN based on the RegiSCAR SJS/TEN validation tool that we informally referred to as “superficial TEN.” Although the BSA and mucosal involvement in these cases were extensive, the epidermal necrosis of the skin seemed to be more superficial and tended to be associated with a better prognosis than those whose necrosis was more typical with the necrosis extending comparatively deeper into the skin. The most obvious clinical difference between the two is the propensity to bleed in the latter group if denuded skin is > 5 cm^2^. This suggests a differential extension of the primary pathology into the dermis. Figure 1 illustrates two “definite” cases of SJS/TEN with comparable BSA involvement at the peak of their disease but different depths of disease extension.

To further support the hypothesis that sometimes the primary pathology in SJS/TEN extends well beyond the epidermis and affects at least progenitor and stem cell populations in affected tissues, two cases of SJS/TEN exclusively managed with supportive care in our unit are highlighted. The first, a previously published case, was a 36-year-old HIV-infected woman of African descent with a CD4+ count of 510 cells/mm^3^ and on zidovudine, lamivudine, and efavirenz for 3 years who desired to conceive. Efavirenz was substituted with nevirapine in her antiretroviral regimen. A week later, she developed a “definite” case of TEN that peaked at 70% BSA. She also developed persistent bilateral corneal perforations despite amniotic membrane transplant. All drugs had been stopped within 48 h of the first symptoms. During her 158-day hospitalization, her skin failed to re-epithelialize despite numerous attempts to skin graft-denuded areas as well as culture and transplant her keratinocytes *in vitro* to promote healing. All the donor sites also failed to heal. Multiple skin biopsies showed lack of epithelial markers. She died of disseminated tuberculosis and septic shock (20).

The second case is a 40-year-old woman of African descent with epilepsy since the age of 12 who presented to us with a “definite” case of TEN, peaking at 40% BSA and uncharacteristically affecting the scalp. She was 24 weeks pregnant with twins. She had started lamotrigine 17 days earlier, having previously been on phenytoin and sodium valproate uneventfully. All drugs were stopped within 24 h of the first symptoms. Her course in hospital was complicated by a miscarriage of both twins 2 days post admission, keratitis, failure to re-epithelialize, and recurrent systemic bacterial infections well into the evolution of her disease. She eventually had extensive full-thickness skin grafting 122 days after the disease onset. This has been complicated by extensive keloid formation in the grafted areas, although she had no history of hypertrophic scarring or keloid formation (21).

In both cases, time to re-epithelialization and duration of hospitalization were considerably longer than averages in large studies with similar BSA (8, 22–25). A delay in the withdrawal of the offending drug, drugs with longer half-lives, preexisting comorbidities, and ethnic background have been suggested associations with prolonged progression and delayed healing in SJS/TEN, the latter a potential proxy for SJS/TEN severity (26–28). As illustrated by these two cases and others in the literature, delayed healing can be associated with different drugs, can occur in any Fitzpatrick skin type, and does not require delayed cessation of the offending drug or HIV infection (26, 29). It is not clear whether scalp involvement, hypertrophic scarring, and/or keloid formation are markers of deeper extension of the primary pathology beyond the epidermis in SJS/TEN.

### Internal organ involvement as an additional marker of Stevens–Johnson syndrome and toxic epidermal necrolysis severity

Bacterial systemic infection (BSI) and septic shock have been shown to be the major causes of intensive care unit admission and death in SJS/TEN (18, 30). In a retrospective Taiwanese study of 150 patients with SJS/TEN, 21% developed disseminated intravascular coagulation (DIC), a marker for BSI. TEN, compared with SJS and SJS/TEN overlap, was significantly associated with the development of DIC, elevated procalcitonin levels, and a 7-fold increase in mortality (31, 32). The organisms isolated from the bloodstream in BSI seem to originate from both the skin and the gut (33–35). In a study of 18 SJS/TEN cases managed in a burns center, there were 11 deaths, six of whom had a postmortem examination. Four of these showed acute ulceration of the esophagus, terminal ileum, and colon ranging from complete denudation to focal ulcerations, becoming a potential source of microbial seeding into the bloodstream. The authors acknowledged that systemic corticosteroids administered to the patients could have caused the ulcers among other possible etiologies (35). These studies support the hypothesis that there may be bacterial dislocation from the gut to the bloodstream in SJS/TEN. The gastrointestinal system (GIT) involvement is further supported by reports of SJS/TEN affecting the esophagus, stomach, small intestines, colon, and the rectum. Apart from visualization on postmortem and scopes, reports of gut perforation, intussusception, bleeding, diarrhea, protein-losing enteropathy, hepatitis strictures, and stenosis following SJS/TEN further support GIT involvement in the disease (35–58).

Multitudes of other studies, case series, and case reports strongly support the involvement of other internal organs in SJS/TEN. Involvement of the respiratory system (RS) can manifest in both the acute or chronic settings. A prospective study of 41 consecutive cases of SJS/TEN found “specific” involvement of the bronchial epithelium in 27% of cases. The authors suggested that this was associated with a worse prognosis (59). Mechanical ventilation was necessary for a quarter of 221 patients with SJS/TEN seen at a French national referral center (60). A retrospective study of 32 SJS/TEN cases found 50% to have abnormal lung function tests during routine follow-up (61). In the published literature, RS involvement following SJS/TEN has been characterized by chronic lung disease, bronchiolitis obliterans, interstitial lung disease, pulmonary air leak syndrome, laryngeal obstruction, and obliterative bronchitis, among others (57, 61–76). The genitourinary system is also not spared in SJS/TEN. Approximately 30% of SJS/TEN cases have been reported to have some form of acute kidney injury, some severe enough to warrant hemodialysis (41, 64, 75, 77–79). Perforation of the uterus, vaginal and introital adenosis, cervical/vaginal adhesions and stenosis, labial synechiae, hydrocolpos, hematometra, hematometrocolpos, and endometriosis are the other reported sequelae of SJS/TEN (64, 75, 80–90). In recent years, the chronic sequelae of SJS/TEN have been recognized and described more systematically. Apart from those just described, other chronic sequelae include eye disease, depression, anxiety, post-traumatic stress disorder, nail abnormalities, pigmentary disorders, scarring, hair loss, pruritus, chronic pain, autoimmune diseases, chronic fatigue, and dental abnormalities (15, 16, 61, 63, 64, 91–93). Perhaps one of the most worrisome recent findings is the higher-than-expected mortality rate among SJS/TEN survivors up to a year after the reaction. A study of 460 patients with SJS/TEN by the RegiSCAR study group found an overall mortality of 23 and 34% 6 weeks and 1 year after the reaction, respectively. BSA was a risk factor for mortality only in the first 90 days, whereas serious comorbidities and age influenced mortality beyond 90 days and up to 1 year after onset of reaction. Even when controlling for comorbid conditions and age, SJS/TEN survivors still have excess mortality compared to the general population (19).

The existing literature suggests that SJS/TEN is a systemic disease with internal organ involvement that can influence not only outcomes but evolution of the disease. The inclusion of acute parameters like heart rate, serum urea, bicarbonate, and glucose in SCORTEN, which were normal in the premorbid state and return to normal in a proportion of survivors, further supports systemic nature and internal organ involvement in SJS/TEN. Internal organ involvement has been shown by numerous studies to impact mortality and morbidity. However, the frequency and severity of individual organ involvement and their impact on overall morbidity and mortality are not clear. Although there have been attempts to develop severity grading systems for systemic involvement in SJS/TEN, with varying degrees of focus on cutaneous and internal organ involvement, these are yet to be validated (25, 94–96).

### Inadequate definition of disease status, progression, cessation, and complete re-epithelialization

Disease progression describes the natural history of a disease, such as pain, or levels of a biomarker such as blood pressure or enzyme levels. There are two main measures of response to a therapeutic intervention in any disease, both dependent on the time course of the disease. The most common is a symptomatic effect equivalent to a shift up or down of the natural history curve. Less common but quite clinically important is a disease-modifying effect equivalent to a change in the rate of disease progression. Both measures can be established using clinical outcomes such as symptoms, or biomarkers such as clinical signs and/or other quantifiable indicators of disease status. To adequately determine disease progression, disease status must be clearly determined at baseline (97). Survival and hospital stay are other examples of measurable outcomes.

In interventional studies designed to halt disease progress, it is necessary to have predetermined biomarkers that correlate with the different stages of the disease as it evolves through the natural history. The same biomarkers can then be used to assess disease status at initiation of therapy as well as its evolution in response to treatment or a placebo. One of the challenges confronting SJS/TEN interventional studies currently is inadequate and often inconsistent definition of disease status and consequently disease progression. A recent systematic review and meta-analysis of systemic interventions in SJS/TEN included three randomized-controlled trials and six prospective, controlled observational studies. The limitations of the included studies identified by the authors include failure to report the time to full skin healing; wide treatment variations across institutions; lack of controlling for confounders; inadequate reporting of baseline comorbidities; and the reliance by clinicians on medical history, clinical morphology, and histopathology, as there are no validated biomarkers to aid in the diagnosis or prognostication of SJS/TEN. The authors recommend all these be addressed to improve the quality of the studies (7). A closer examination of the individual studies highlights the variation in endpoints and a generally inadequate definition of these endpoints in even the most robust of interventional studies in SJS/TEN. Other than mortality, endpoints included change in prostration (level of tiredness or weakness); fever; duration of progression of skin detachment; BSA stabilization; arrest of disease progression; beginning and completion of re-epithelialization; recovery velocity index using a severity-of-illness score developed by the authors; illness auxiliary score that includes modified SCORTEN parameters; and a simplified acute physiology score. Apart from variable endpoints, most of the studies do not fully describe these endpoints in a reproducible fashion (25, 95, 96, 98–102).

## Potential future research directions

### Imaging as a global assessment of Stevens–Johnson syndrome and toxic epidermal necrolysis severity

Positron emission tomography (PET) is a non-invasive molecular imaging tool that provides tomographic images and quantitative parameters of perfusion, cell viability, proliferation, and/or metabolic activity of tissues. These images result from the use of different substances of biological interest (sugars, amino acids, metabolic precursors, hormones) labeled with positron-emitting radionuclides. A combination of important functional information provided by PET with morphological detail provided by computed tomography (CT) as PET/CT provides clinicians with a sensitive and accurate one-step whole-body diagnostic and prognostic tool. Fluorodeoxyglucose (FDG) is a radiolabeled analog of glucose and is taken up by cells *via* the first stages of the normal glucose pathway and trapped inside cells with high glycolytic activity. FDG uptake is quantifiable and correlates with metabolic activity, providing useful information on disease severity, disease progression, and therapeutic response (103). FDG-PET/CT has been used successfully to identify, localize, and quantify inflammation *in vivo* in an array of inflammatory conditions affecting the eye, RS, GIT, GUT, and the cardiovascular system. It is a useful tool to detect metabolic responses in infectious processes and other inflammatory conditions (104). The spectrum of clinical diseases on which FDG-PET/CT has shown utility includes connective tissue diseases, vasculitis, arrhythmias, arteriosclerosis, aneurysm detection and progression, sarcoidosis, amyloidosis, psoriasis and psoriatic arthropathy, malignancies, neuritis, encephalitis, eye tumors, myositis, arthritis, osteomyelitis, osteonecrosis, osteitis, transplant rejection, inflammatory bowel disease, hepatitis, glomerulonephritis, lymph node assessment, hidradenitis suppurativa, tuberculosis, and deep fungal infections (105–118). We have used FDG-PET/CT in an ongoing study to determine internal organ involvement and disease severity in patients with SJS/TEN during the acute stage and a later time point. Our preliminary data show very promising proof-of concept results that demonstrate FDG-PET/CT as relatively non-invasive methods of identifying and quantifying tissue involvement in SJS/TEN beyond the skin.

### Artificial intelligence as an aid in the Stevens–Johnson syndrome and toxic epidermal necrolysis disease status

Artificial intelligence (AI) is a general term that implies the use of a computer to model intelligent behavior with minimal human intervention. The application of AI in medicine has two main branches, namely, virtual, and physical. The virtual component is represented by deep learning (DL), a subset of machine learning (ML) that is represented by mathematical algorithms that improve learning through experience. AI’s goal is to build algorithms (“models”) that perform tasks that are considered to require intelligence or training, such as recognizing objects or diseases in images. Traditionally, algorithms are built that can perform image classification tasks by first creating feature detectors (e.g., this is a round spot, this is the color of that spot), then using handcrafted prediction rules (e.g., size > 3 mm, color varying across the spot) to make classifications. However, this can be difficult and the models may be brittle (e.g., the spot detection fails, or the color quantification fails because the lighting is different, or the size detection fails because the skin is a variable distance from the camera).

There are three types of ML algorithms, namely, (1) unsupervised (ability to find patterns), (2) supervised (classification and prediction algorithms based on previous examples), and (3) reinforcement learning (use of sequences of rewards and punishments to form a strategy for operation in a specific problem space) (119). ML is a set of computational techniques to build algorithms that learn from data (i.e., “training data”) instead of being engineered to detect specific features. Dermatology, as a predominantly visual specialty, is suitable for ML because there is sufficient complete training data in the form of clinical images. This is more accurate than handcrafted approaches that input data handpicked by the data scientist into the model. For example, by training an algorithm using tens or hundreds of thousands of images of SJS/TEN across a variety of lighting conditions and backgrounds, the algorithm can learn the morphologies that correspond to the disease more accurately.

Deep learning is the dominant AI technology that leverages complex data, such as images, through artificial neural networks that learn complex mappings between inputs (e.g., images) and outputs (e.g., diagnoses) without explicit human engineering. The model self-learns features from the input, such as visual patterns, that are most relevant for predicting the output. In many settings across medical specialties, DL matches healthcare professionals in detecting disease from medical imaging (120). AI is progressively being integrated into clinical care of skin diseases. An AI system has already been approved for the European market as a medical device for the management of melanoma. The device was shown to perform comparably with dermatologists who reviewed text and clinical images of melanomas in a setting simulating store-and-forward teledermatology (121). A DL system for diagnosis of early SJS/TEN images vs. non-severe cutaneous adverse drug reactions based on imaging of the individual lesions has recently been developed. This was shown to perform significantly better than all 10 board-certified dermatologists and 24 trainee dermatologists involved in the study (122). AI offers a significant opportunity to harmonize SJS/TEN disease status and endpoints across studies.

### Biomarkers as tools for measuring disease severity

Previous SJS/TEN studies have mostly focused on genetic biomarkers and others to predict mortality. There have been much fewer studies focusing on biomarkers to monitor severity, progression, and response to therapy during the acute stages of SJS/TEN and how these correlate with long-term morbidity and delayed mortality. Biomarkers that have been studied either singly or in combination in SJS/TEN include procalcitonin (32); granulysin (123); IFN-g (124); interleukin (IL)-8 and granzyme B (125); endocan, tumor necrosis factor-α, vascular endothelial growth factor, and C-reactive protein; serum IL-17 (126); complement components (127); alarmins like the heterodimeric form of S100 calcium-binding protein A8 and S100 calcium-binding protein (A9 S100A8/A9) (123); chemokines like CXCL9/MIG and CXCL10/IP-10 (124); antimicrobial peptides like LL-37 (128); exosomal nucleic acids like *miR-375-3p* (129); plasma lipid profiles (130); renal functions (78, 79); neutrophil:lymphocyte ratio; and C-reactive protein:albumin ratio (131).

Systematic pattern comparison of biochemical, inflammatory, hematological, and immune biomarkers in SJS/TEN cohorts stratified by severity and mortality may enable sufficient discrimination to warrant inclusion in risk stratification models. In these types of studies, lack of clinically or statistically significant differences does not necessarily imply a lack of association with the outcomes being measured (132). Thus, it is important to have a low threshold for biomarker inclusion in study designs and building predictive risk stratification models.

### Development of consensus on definitions of endpoints in interventional studies

Significant coherence has emerged among the leading researchers in SJS/TEN over the last decade. Numerous meetings that brought together international experts and researchers have successfully been convened in Asia and North America. The meetings have been effective in collating together the current body of knowledge, allowing closer collaboration among researchers and mapping research agenda on SJS/TEN. Some of the highlighted gaps, including definitions of disease severity, progression, and complete re-epithelialization, can be addressed in these meetings of experts and consensus reached. Similarly, researchers at the forefront of biomarker research can collectively study the most promising biomarkers and map research direction. This would further allow sharing of progress made, including negative findings that would otherwise not make it into publication. Unless these and other similar collaborative efforts are adopted, the proposed international multicenter pharmacotherapeutic interventional studies may not provide robust evidence (133).

### Limitations

The limitations of this work include the use of individual case reports to highlight the gaps in current practice that may be outliers and not generalizable to all patients with SJS/TEN. Additionally, these are proposals that may not be successfully implemented in real-life settings.

## Conclusion

There are gaps that need to be urgently addressed in SJS/TEN research. There is an urgent need for reproducible methods of measuring disease severity that are sensitive to changes induced by therapeutic interventions and that more accurately predict outcomes beyond the acute stage by including the systemic and internal organ effects of SJS/TEN. Potential solutions include consensus on definitions, advances in diagnostic imaging and biomarker assessment, and development of AI platforms for the detection and monitoring of disease.

## Data availability statement

The raw data supporting the conclusions of this article will be made available by the author, without undue reservation.

## Ethics statement

The studies involving human participants were reviewed and approved by Human Research Ethics Committee, University of Cape Town. The patients/participants provided their written informed consent to participate in this study. Written informed consent was obtained from the individual(s) for the publication of any potentially identifiable images or data included in this article.

## Author contributions

The author confirms being the sole contributor of this work and has approved it for publication.
